# Climate Change and the Future of California's Endemic Flora

**DOI:** 10.1371/journal.pone.0002502

**Published:** 2008-06-25

**Authors:** Scott R. Loarie, Benjamin E. Carter, Katharine Hayhoe, Sean McMahon, Richard Moe, Charles A. Knight, David D. Ackerly

**Affiliations:** 1 Nicholas School of the Environment & Earth Sciences, Duke University, Durham, North Carolina, United States of America; 2 Department of Biological Sciences, California Polytechnic State University San Luis Obispo, San Luis Obispo, California, United States of America; 3 Department of Geosciences, Texas Tech University, Lubbock, Texas, United States of America; 4 Jepson Herbarium, University of California Berkeley, Berkeley, California, United States of America; 5 Department of Integrative Biology, University of California Berkeley, Berkeley, California, United States of America; Monterey Bay Aquarium Research Institute, United States of America

## Abstract

The flora of California, a global biodiversity hotspot, includes 2387 endemic plant taxa. With anticipated climate change, we project that up to 66% will experience >80% reductions in range size within a century. These results are comparable with other studies of fewer species or just samples of a region's endemics. Projected reductions depend on the magnitude of future emissions and on the ability of species to disperse from their current locations. California's varied terrain could cause species to move in very different directions, breaking up present-day floras. However, our projections also identify regions where species undergoing severe range reductions may persist. Protecting these potential future refugia and facilitating species dispersal will be essential to maintain biodiversity in the face of climate change.

## Introduction

The California Floristic Province has over 5500 native plant taxa; 40% of them are endemic, that is, their entire native distributions are within the Province [1]. (By taxa, we mean distinct species, subspecies, or varieties, and we use “species” hereafter for simplicity [2].) Models project that California's temperature and rainfall will change considerably in this century [3]. Here, we use observed data on species' distributions and present-day climate to build multiple bioclimatic models. We then apply these models to project changes in endemic species' range sizes, distribution and diversity under future climate scenarios.

Empirical examples of species' range shifts resulting from climate change have been recorded for numerous taxa [4]–[5]. Projecting future changes is a crucial step towards planning for and mitigating the impacts of climate change on biodiversity [6]. Most previous attempts have focused on small subsets of species [7]–[9] or vegetation types [10]–[11]. They incorporated varying degrees of data on physiology and dispersal. A small number of related studies have focused on estimating changes in biodiversity [12]–[13]. As we describe in the methods, biodiversity studies must limit themselves to species subsets restricted to the region – such as endemics [14]. Sparse flora-wide data on physiology and dispersal has meant that studies across floras have included simplified treatments of individual species' biology.

A recent study of two California oak species projected significant range reductions for both species [7]. In southeastern California, a study of *Yucca brevifolia* that included physiological responses to increased CO_2_ levels projected a slight decrease in range size [15]. Analysis of the responses of vegetation types in California to climate change projected decreased coniferous forests in the northwestern part of the state and increases in broadleaf vegetation [16]. In Eastern North America, models for 80 tree species project range expansions for approximately 30 species and an equal number of range contractions. In that study, the centroids of nearly half of the species were projected to move at least 100 km to the north [17].

Outside of North America, regional studies have addressed both range shifts and potential levels of extinction in the face of climate change. Studies of the Proteaceae in the Cape Floristic Province – another Mediterranean hotspot — estimate that this group may lose up to 20% of the species considered [8], [18]–[19]. A study of 975 endemic plant species in southern Africa projected that the Mediterranean climate portion of the study area will lose the highest proportion of species [20], while flora-level studies from Europe have projected that as many as half of the species studied will be threatened [12]–[13], [21].

Currently, there are no published assessments of potential impacts of climate change on regional endemic floras for any part of North America. California is particularly well suited to such a study, as it has high endemic plant diversity and the quality of plant distribution and climate data across the region are excellent. California also provides an interesting case study because of its topographic complexity, extensive urban and agricultural land use, and Mediterranean climate characterized by distinctive rainfall and temperature patterns.

We assess 8 different potential scenarios for the future of the California flora in the face of climate change. These are the combinations of three pairs of possibilities. First, we compared two projections of future emission levels from human activities. One is higher, with global CO_2_ emissions reaching almost 30 GtC per year, or 4 times present-day levels, by 2100 (SRES A1FI) while the other emission scenario is lower, with CO_2_ emissions rising slightly by mid-century before dropping to below present-day levels by the end of century (SRES B1) [22]. By 2100, global atmospheric CO_2_ levels reach 550 and 970 ppm under the lower and higher emissions scenarios, respectively. Second, we compared projections centered 80 years from now (2070–2099) from two global climate models with higher and lower sensitivities to atmospheric greenhouse gas levels. The U.K. Meteorological Centre's Hadley Centre Coupled Model version 3 (HadCM3) model [23]–[24] is moderately sensitive to increases in emissions, while the DOE/NCAR Parallel Climate Model (PCM) is less sensitive [25]. Third, we explored two distinct and widely used dispersal scenarios: one where plants exhibit unrestricted movement to new locations, and one with no movement [26], [13], [18].

Projecting the impacts of climate change to an entire endemic flora is complicated by scarce and variable distribution data. Studies conflict on how many geo-referenced specimens are necessary to obtain robust species projections [27], [28]. Including poorly known species risks biasing projections of biodiversity patterns if the error is directional. In contrast, poorly known species may have smaller ranges, and small ranged species are known to be more vulnerable to extinction [29]. Excluding such species may be equally inappropriate.

A recent study recommends using Maxent and at least 30 non-validation specimens for robust species projections [28]. Following these recommendations, we model and evaluate the 591 out of 2387 California Floristic Province endemic species that have at least 42 specimens using Maxent [30]. Specimen records were obtained from the Consortium of California Herbaria, a centralized portal accessing over 959,000 specimens from 16 herbaria [31]. To address whether poorly known species tend to have small ranges, we compile an independent dataset of range maps for each species to compare with the number of specimens. (We refer to these as TJM1 range maps, see Materials and Methods.)

To assess whether excluding poorly known species biases diversity patterns, we build a multilevel generalized linear model (MLGLM) [32] incorporating all 2069 species with at least 2 specimens. This model simultaneously estimates relationships between the probability of a plant being found in a location, and climatic variables. It does so both at the level of each species as well as the entire flora. The hierarchical structure of this model gives an unbiased predictor of climate influences on presences, and allows poorly known species to draw inferential strength from the flora as a whole [33]. As a result, the model is informed by data from all species, but the influence of poorly known species is properly weighted against the flora. We then compare biodiversity patterns from this hierarchical approach with Maxent projections from the best known 591 species.

To summarize the impacts of climate change on the California flora and to compare the projections with other studies, we ask four questions. First, where will endemic species diversity be most influenced by climate change? Second, if species are permitted to move, where will they go? Third, how do we project range sizes to change? Fourth, where do we expect future refugia — locations where species at risk from climate change will persist under future climates? To date no studies have mapped the locations of such refugia.

## Results

### Study area

**Figure 1:**
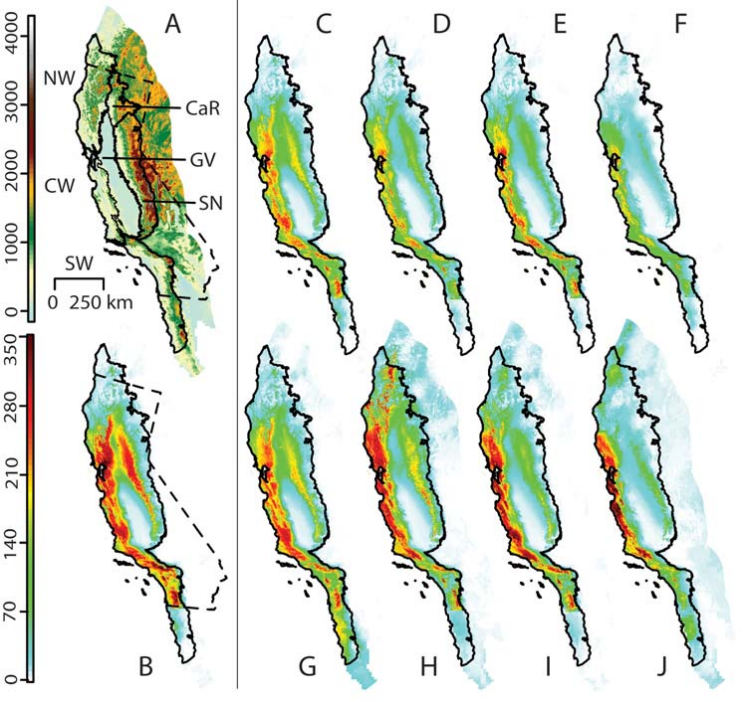
Study area and Maxent diversity projections of the best known 591 species. (A) The province divided into six floristic regions (solid lines): Northwestern California (NW), Central Western California (CW), Southwestern California (SW), the Cascade Ranges (CaR), the Great Central Valley (GV), and the Sierra Nevada (SN). The province includes most of California (dashed line) and portions of Oregon and Mexico. We include a surrounding buffer of equal area (colored areas outside solid line). Colors represent elevation in meters. (B) Projected present diversity. (C–J) Projected diversity 80 years from now modeled with increasing amounts of future climate change: (C–F) Plants cannot disperse. (G–J) Plants can disperse to all suitable areas. (C, F, G, H) Simulations based on the lower sensitivity PCM model. (E, F, I, J) Simulations based on the higher-sensitivity HadCM3 model. (C, E, G, I) Lower emissions scenario (B1). (D, F, H, J) Higher emissions scenario (A1FI).

The California Floristic Province (Fig. 1A, solid line) occupies approximately 310,000 km^2^. It is ecologically and climatically delimited and its flora is both rich and well studied [34]–[36]. The six constituent floristic regions — Northwestern California, Central Western California, Southwestern California, the Great Central Valley, the Cascade Ranges, and the Sierra Nevada — encompass elevations from 200 m below sea level to about 4,000 m. The Province includes almost all of California (Fig. 1A, dashed line), except its deserts and the northeastern Modoc Plateau, as well as adjacent parts of Mexico and Oregon. The study area for this paper includes the entire California Floristic Province and a surrounding area of equal size in the form of an approximately 200 km wide buffer (Fig. 1A, all colored areas).

### Diversity change

We created diversity maps by summing modeled species distributions as is commonly done in Gap analysis [37] and biodiversity studies [38]. First, we present Maxent projections from the 591 species with the most distribution data. We then compare these projections with approaches that include poorly known species.

Based on these 591 species, we project present-day endemic diversity to peak at 340 species per km^2^, with the highest concentrations from southern Northwest California through most of Central Western California and in the foothills of the Sierra Nevada (Fig. 1B). These results correspond to previous descriptions of patterns endemic diversity in the California Floristic Province [39].

Our models yield projections of future diversity under a range of climate change scenarios (Fig. 1, C through J). We contrast scenarios where species cannot move — and so their ranges can only shrink (Fig. 1, C through F) — with those where species are allowed unrestricted movement to new areas that satisfy their climatic constraints (Fig. 1, G through J).

Under the highest level of climate change examined here (mid-high climate sensitivity and higher emissions, as represented by HadCM3 A1FI projections), with the assumption of no dispersal, we project peak diversity to drop as low as 247 species per km^2^ (Fig. 1, F). In contrast, under relatively low amounts of climate change (low climate sensitivity and lower emissions, as represented by PCM B1 projections), and allowing for dispersal (Fig. 1, G and H), diversity increases across extensive areas, particularly the northern coasts. As expected, the worst-case scenarios come from the higher sensitivity simulations (HadCM3: Fig. 1, E, F, I, J) compared to the lower sensitivity simulations (PCM: Fig. 1, C, D, G, H). Similarly, projections based on the higher emissions scenario (A1FI: Fig. 1, D, F, H, J) alter diversity more than those based on lower emissions (B1: Fig. 1, C, E, G, I). Dispersal greatly buffers climate impacts on total diversity, as species gains may partially or wholly offset losses at a local level (Fig. 1, C–F vs. G–J).

Across all scenarios, the general trend is that diversity shifts towards the coast and northwards. Coastal areas, especially Northwestern California and Central Western California, are presently rich in species. Even under significant climate change, they will continue to be so. In contrast, the foothills of the northern Sierra Nevada are extremely vulnerable to species loss. Under scenarios that allow dispersal, the areas that straddle the California-Oregon border also become rich in species — as expected from northward dispersal.

### Diversity change and poorly known species

**Figure 2:**
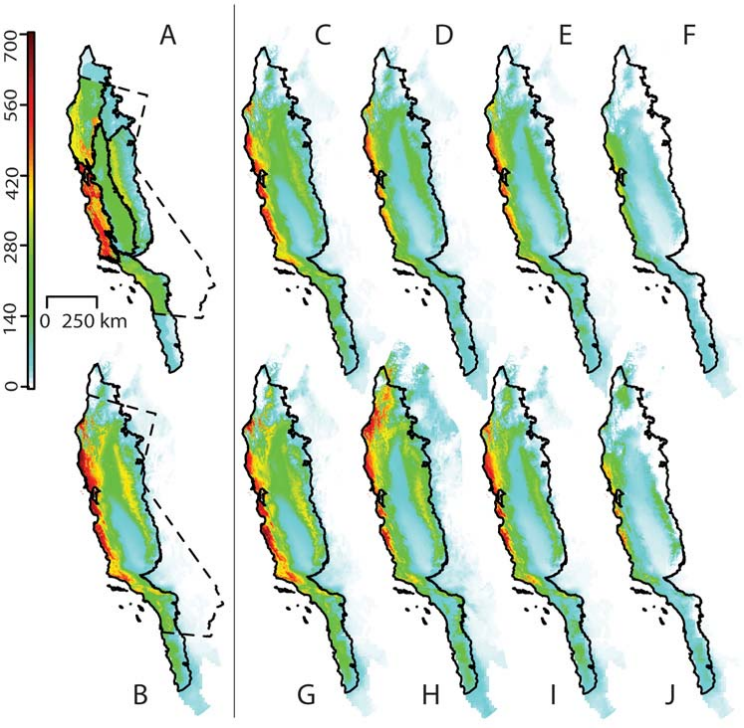
(A) Present diversity from range maps for all California Floristic Province endemic species (2387). (B) Projected present diversity from the Multi-level Generalized Linear Model for all species with >2 specimens (2068). (C–J) Projected diversity 80 years from now modeled with increasingly increasing amounts of future climate change: (C–F) Plants cannot disperse. (G–J) Plants can disperse to all suitable areas. (C, F, G, H) Simulations based on the lower sensitivity PCM model. (E, F, I, J) Simulations based on the higher-sensitivity HadCM3 model. (C, E, G, I) Lower emissions scenario (B1). (D, F, H, J) Higher emissions scenario (A1FI).

The number specimens and range size derived from the TJM1 range maps were positively correlated (ρ = 0.49). Summed range maps for all 2387 endemic species indicate that species richness peaks at 621 species (Fig. 2A).

Figure 2B shows present diversity projections of 2068 species from the MLGLM generalized linear model. Diversity patterns in figure 2B are similar to those in figure 2A except that the range map derived diversity is lower in Northwestern California and Southwestern California. The patterns in figure 2B differ from the Maxent projections in that diversity is lower in the Sierra Nevada and Southwestern California and higher in coastal Northwestern California.

Changing patterns of diversity projected from the multilevel model are very similar to the patterns of diversity projected from Maxent. In general, diversity shifts towards the coast and northwards, and the degree depends on the dispersal assumptions, emission scenarios, and the sensitivity of climate simulations. The following results on species movement and range size change are from Maxent projections of the 591 best known species.

### Species movement

Changes in diversity reflect the overall consequences of local extirpation and species dispersal. These patterns do not address the potential fate of individual species. For that reason, we also examined individual species fate in terms of projected geographic shifts in species' mean elevation, range centroid, and percent change in range size. In high emission scenarios (A1FI) with dispersal, we project species range centroids to shift by an average of up to 151 kilometers (see Fig. S1).

**Figure 3:**
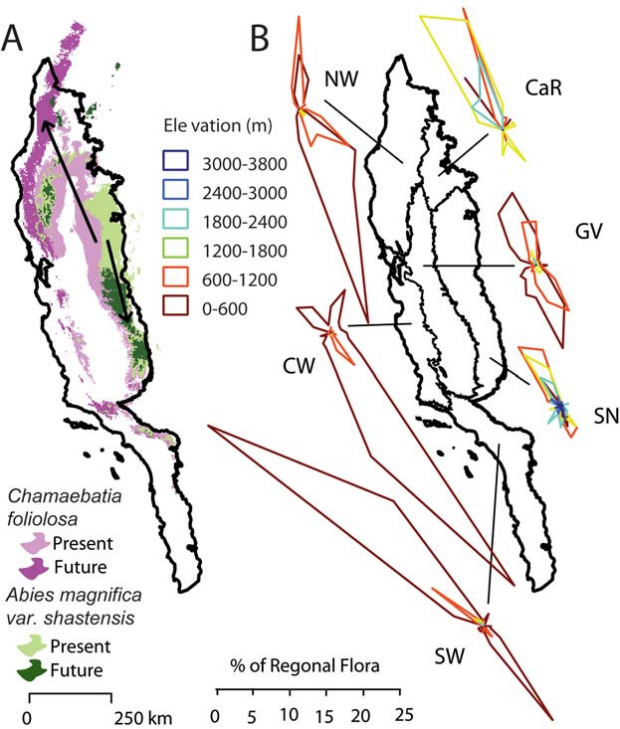
Movement of species geographic centroids based on HadCM3 simulations using the A1FI emission scenario 80 years in the future and assuming species can move. (A) Two representative species that have adjacent present ranges (lighter colors) and are projected to move in opposing directions (arrows and darker colors). (B) Projected centroid movements for all species. Individual polar plots group species by the floristic region in which their centroid originates. Within each plot, species are grouped by the elevation in which their centroids originate. The magnitude of the directions represents the percentage of the regional flora moving in each direction.

As one might expect, species tend to move to higher elevations and often northward (see Fig. S2). Interestingly, these trends result in divergent projections for elements of the flora. Given California's geography, movement to higher elevations often means taking a *southward* path. Figure 3A illustrates two representative species that presently have essentially adjacent ranges. In the future, we project their ranges to be widely separate, with one moving south to higher elevation regions of the Sierra Nevada, and the other moving north and towards the coast. Figure 3B illustrates the broader consequences, based on analysis of the centroids of the species' ranges. They are ecologically dramatic. Within the six major regions, substantial numbers of species move in diametrically opposite directions — typically north of northwest, and south of southeast. In the Cascade Ranges and the Sierra Nevada, species at high elevations tend to move south to higher elevations. Those at lower elevations, like those in other regions, are a mix of species, some of which move south and others that move north. (See Fig. S3 for scenarios not shown here).

The results shown here are for the largest projected changes in temperature (HadCM3, A1FI), allowing dispersal. We obtain similar patterns under lower projections of climate change and without dispersal (when species ranges can only shrink). In short, even relatively moderate projections suggest that climate change has the potential to break up local floras, resulting in new species mixes, with consequent novel patterns of competition and other biotic interactions.

### Range size change

As in previous studies in Europe and southern Africa, we project both reductions and increases in range sizes, depending on the degree of climate change and the abilities of the species to disperse [12]–[13], [20]–[21]. Under scenarios without dispersal, we project that up to 66% will experience >80% reductions in range size. The magnitude of variability in range size change forecasts is comparable with a recent study based on global vegetation modeling, rather than species-based models [40]. (See Fig. S4 for summaries of range size change).

**Figure 4:**
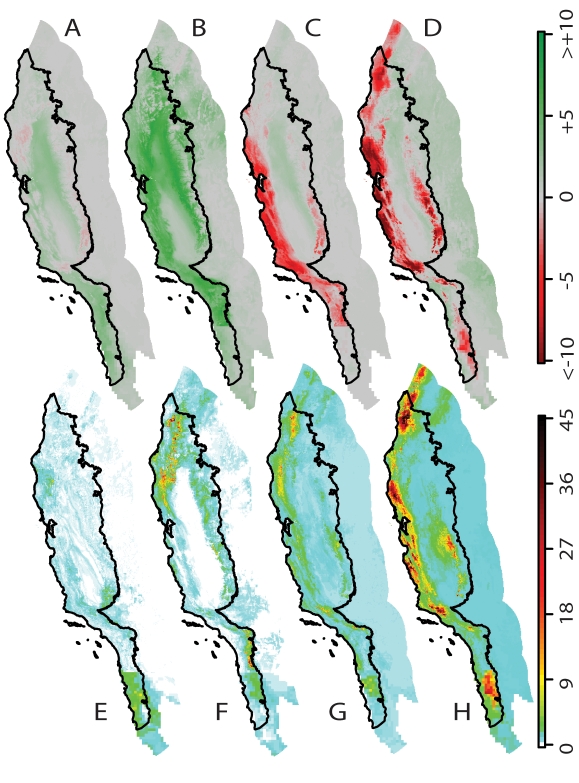
Future patterns of range size changes across increasing levels of climate change in which species can move. (A - D) Percent geometric mean change in range size (Future/Present with colors stretched from a <-10% decrease to a >10% increase). (E - H) Diversity of species gains (future diversity with migration minus future diversity without migration) for the quarter species suffering the largest range contractions. (A, B, E, F) Simulations based on the lower-sensitivity PCM model. (C, D, G, H) Simulations based on the higher-sensitivity HadCM3 model. (A, C, E, G) Lower emissions (B1). (B, D, F, H) Higher emissions (A1FI).

Figure 4 shows the geographic patterns of change in range size. Figure 4, A through D, maps the geometric mean of the changes in range size for species projected to occupy each pixel on the map, for scenarios with dispersal. The minimum mean decrease in range size is −58% in Central Western California in the HadCM3, A1FI scenario. The maximum mean increase in range size was +35% in the foothills of the Great Central Valley in the PCM, A1 scenario. We stretched the colors from −10% to +10% in order to show the majority of more moderate range size changes.

Green areas are dominated by species with expanding ranges. Red areas harbor shrinking species; they are climate change refugia for the species that a future generation of biodiversity managers may classify as “threatened”. In the future, the lower sensitivity simulations (PCM: Fig. 4, A–B) project extensive areas dominated by species with expanding ranges, particularly the more Mediterranean PCM, A1FI scenario (Fig. 4B). In these scenarios the southern Sierra Nevada and the mountains of Northwestern California harbor shrinking species. In the higher sensitivity simulations (HadCM3: Fig. 4, C–D) these areas are joined by the coastal mountains of Northwestern California and Central Western California which are dominated by species projected to suffer range reductions.

The red refugia in Figure 4 A–D combine species contracting into their current ranges and shrinking species dispersing into new areas. Figure 4 E through H maps out the gains (future diversity with dispersal minus future diversity without dispersal) of the quarter of the species undergoing the greatest range reductions. The potential for these areas to act as refugia depends greatly on whether species are able to disperse into them.

## Discussion

### Model projections of diversity, range size, and species movement

The projections of diversity change are comparable with other studies from Africa and Europe [12]–[13], [20]–[21]. As in these studies, model projections depend greatly on future climate simulations, emission levels, and dispersal scenarios. As in studies in the South African Cape, we found that species losses were disproportionately clustered in montane areas as opposed to lowlands [19]. We also project that these montane areas, particularly the coastal mountains, are where large number of species will persist.

The magnitude of our range centroid shifts is similar to those reported for Eastern North American trees [17]. Kueppers *et al.* projected that the range centroids of the two California oak species they considered would shift northwards [7]. Likewise, Lenihan *et al.* projected broadleaf forests – which include oak woodlands – to move north into what are now chiefly coniferous forests [16]. We projected large numbers of species – including these oaks – to behave similarly to these prior projections as they expand into Klamath Mountains on the California-Oregon border and recede from the center of the state. This similarity is despite our use of climate simulations that project greater increases in temperature and decreases in precipitation than those used by Kueppers *et al.* Across the entire flora, however, we project that large numbers of species will shift south as they cluster around the coastal mountains of southern California. Kueppers *et al.* projected the two oak species ranges to contract. As reported by other flora wide studies [20]–[21], our projections of range size change vary greatly based on future climate simulations, emission levels, and dispersal scenarios. Under all scenarios explored here except the PCM simulation with A1FI emission levels, we also project the ranges of these two species of oak will contract.

### The influence of poorly known species

The positive correlation between range map derived range size and number of museum specimens raises legitimate concern that excluding poorly known species may bias the results. From the comparison of the Maxent results from 591 species and the MLGLM results from 2068 species, we did not find the exclusion of these poorly known species to influence the general patterns of projected present and future biodiversity. These results suggest that the patterns of projected biodiversity presented here are robust despite the exclusion of poorly known species.

### Model uncertainty and performance

The bioclimatic models implemented in this study make a number of simplifying assumptions that may bias the projections [41]–[42]. The models ignore several factors that would exacerbate the projected impacts of climate change. These include specialization to restricted soil types [43], the spread of invasive species [44], local adaptation of populations within species, and genetic constraints on evolutionary response to climate change [45]. On the other hand, resilience of established plants and seed banks [46], differing population responses at range margins [47], and adaptive evolutionary responses might mitigate the influence of climate change. Effects of wildfires, projected to increase in the future [48], are uncertain. Both climate change and uncertain changing land-use patterns will impact species distributions [49]. It is uncertain what the cumulative effect of these dual threats on species will be [50]. Preliminary modeling efforts to incorporate current and future land-use estimates showed that reduced habitat from increased urban and agricultural development led to further declines in projected diversity, but did not qualitative alter the outcomes presented here.

A key simplifying assumption is the “equilibrium postulate” [51]–[52] that species' current ranges are in equilibrium with their environment and there are no time lags on the influence of past climate on current species distributions [53]. While this may not be the case in parts of California where plant ranges are still responding to the post-glacial conditions, human induced climate change is projected to be far greater than post-glacial change. Thus, it is likely that species responses to human induced climate change will far outweigh any post-glacial response. Another concern is that if drivers not considered in these models are correlated with the climate data, we may wrongly attribute species distributions to climate tolerances. Furthermore, our models ignore the influence of species interactions on plant ranges [54]. Exploring these simplifying assumptions represent important avenues for future research.

As described in the materials and methods, we evaluate Maxent projections for the current time period using two widely-used statistics calculated from a set of evaluation specimens independent from the specimens used to train the models [28], [55], [56]. While these evaluation methods indicate that the models performed very well, they do assume that models that predict current ranges well will also predict future ranges well. Recent studies have questioned this assumption [57]. Different models with equivalent current projections may project very different future ranges based on how those models interpolate new climate combinations not represented in the current climate data [58]. Likewise the evaluation procedure does not incorporate uncertainty in future climate projections or species dispersal. These sources of uncertainty may be significant when, as in the case of rainfall, the climate variables are particularly important determinants of plant distributions [59].

### Management considerations

These results present a sobering picture of the potential impacts of climate change on California's diverse and distinctive flora. The severity of projected impacts is closely linked to the magnitude of climate change. That, in turn, depends crucially on human emissions of greenhouse gases over the next few decades. The projected impacts are also very sensitive to the potential rate of plant movement, and rapid dispersal could mitigate much of the impact on individual species and overall diversity. However, rapid movement by natural dispersal is unlikely on a century time-scale, except for weedy species with short generation time and highly dispersable propagules. Human assisted dispersal must be considered as a critical component of conservation and biodiversity management in the next century.

The results of this study present a dilemma for conservation planning in the face of climate change. Future diversity will likely peak along the coast and to the north of its present concentrations (Fig. 1). These areas are sensible priorities for conservation. Some areas of high diversity, however, will be comprised of species expanding their ranges, and these species may not represent important targets for conservation efforts. Areas that are projected to harbor species with shrinking ranges, on average (Fig. 4, A–D), include many mountainous areas scattered across the study area. We identify these areas as refugia that may disproportionately contain the most “threatened” species. These “future refugia” present valuable opportunities as conservation targets. They may protect significant components of biodiversity into the next century. The number of species projected to survive in these refugia (Fig. 4, E through H) depends critically on the ability to disperse, highlighting the importance of landscape connectivity and potential restoration in the face of increasing urbanization, land use change, and disturbance.

## Materials and Methods

### Distribution data

We compiled geo-referenced specimens from the Consortium of California Herbaria [31] (accessed April 27, 2008) for the 2068 endemic species with at least two specimens. The average number of specimens per species was 37 with a maximum of 495. Of these 2068 species, 591 had at least 42 specimens (a minimum of 31 for model training and 11 for model evaluation).

Additionally, we built range maps for each species from The Jepson Manual, 1^st^ edition (TJM1) [2], a flora that provides distribution information for every vascular plant species found within the state of California. The Jepson Manual divides the California Floristic Province portion of the state into 28 polygons called subregions. Experts recorded each species as present or absent in each subregion. In addition, experts assigned lower and upper elevation limits to each species. We hand drew the Oregon and Baja California portions of range maps for 508 species that range outside state of California with data from the Oregon State University Herbarium [60] and the Flora of Baja California [61]. The range maps, which we refer to as TJM1 range maps, are the intersection of the elevation limits and the subregion polygon using a widely used digital elevation model [62]. See Table S1 for a list of number of specimens for each species and the TJM1 range map derived range size.

### Current climate data

We created four largely independent climate variables to represent present climate, derived from average monthly mean temperature and monthly total precipitation from the 1 km resolution DAYMET 1980–1998 mean climate database (www.daymet.org) [63]. As DAYMET does not cover Mexico, baseline climate data for Baja California portions of the study area were derived from an1/8th degree climate baseline database. DAYMET and the 1/8th degree climate baseline database are both geographic interpolations of climate station data with two principle differences. They are interpolated at different spatial resolutions and the network of stations in Mexico is generally sparser than in the US. For each dataset, we averaged the same variables – monthly mean temperature and monthly total precipitation – across the same time period, 1980–1998 [64].

The four climate variables were the first two axes of two principal components analyses (PCA), one based on the 12 monthly mean temperatures and one on the 12 monthly precipitations, respectively (Fig. S5, A–D). We used the *prcomp* function in R to perform the PCA. The first two axes comprised 69% and 20% of the variation in monthly temperatures and 48% and 21% of the variation in monthly precipitations. In each case, the first PCA axis approximated the magnitude (mean temperature and total precipitation) and the second axis the seasonality in temperature and precipitation (Fig. S5, E–H). For each PCA, the two axes are orthogonal by definition. Correlations among axes between the two PCAs ranged from −0.53 to 0.40. Orthogonal PCA axes have two principle advantages. They optimally summarize month-to-month variation in climate, and they eliminate interactions among correlated variables. The disadvantage of PCA axes is that they can be difficult to interpret. We selected the two independent PCAs, rather than a single PCA across all climate variables, to balance ease of interpretation of temperature and precipitation with the statistical advantages of working with largely orthogonal variables.

### Maxent Models

For each of the 591 best known species, we used Maxent (version 2.3) [30] to model habitat suitability from the four climate variables. We used the default convergence threshold (10^−5^) and maximum number of iterations (500) values. We withheld 25% of the specimens for model evaluation. We let Maxent select both suitable regularization values and functions of climate variables automatically, which it achieves based on considerations of sample size. Maxent outputs a continuous index, ranging from 0 to 100, an indicator of relative suitability for the species, based on the principle of maximum entropy, as constrained by the input occurrence data. Choosing an appropriate threshold must balance errors of commission and errors of omission. We used the widely adopted method of thresholding the point on the reciever operating characteristic curve where the sum of the sensitivity and specificity is maximized (see below).

### Maxent model evaluation

We used the test specimens to evaluate the performance of the Maxent projections using two widely used statistics that are recommended when evaluation absences are unavailable. The first was the area under the receiver operating characteristic curve [65] modified for use with a presence only test data [30]. This statistic measures model performance by plotting the sensitivity values – the true positive fraction of test points – against 1-specificity – the false-positive fraction for all available probability thresholds [53]. The average value of the statistic, which can range from 0.5 (random) to 1.0 (perfect discrimination) was 0.95.

The second statistic was prediction success, the percentage of positive evaluation occurrences correctly classified as positive [28]. This statistic is threshold dependent and uses the binary distributions. The average prediction success was 0.93. See Table S2 for evaluation statistics for each of the 591 species.

Despite being statistically defensible, the chosen thresholds produced diversity maps that exceeded the diversity calculated from the TJM1 range maps. Since range maps are known to overestimate range size by over interpolating patchy species distributions [38], range map derived diversity should provide an upper-bound on diversity estimates. This serves as a reminder that distribution modeling with presence only data is inherently qualitative [66]–[67]. We caution against over interpreting the magnitude of the biodiversity projections. Comparisons with the multilevel model, however, indicate that the spatial patterns are robust.

### Multi-level Generalized Linear Model

Unlike Maxent, generalized linear models require presence and absence data. To generate absence data for each of the 2068 species with at least 2 specimens, we generated a random (from 1 to 54) number of informed pseudo-absence data by randomly sampling points from outside the species' range map. See Table S2 for a list of the number of pseudo-absences for each species. We chose this configuration to maximize the variability among presence/absence ratios for the species to aid model convergence. We used the presence/absence data from these 2068 species to build a hierarchical model of the probability of species occurrence as a function of the climate data.

The multi-level model has two levels: a flora level and an individual species level. At the flora level, the model estimates 9 parameter values for a data matrix consisting of an intercept, linear versions of the four climate variables, and quadratic versions of the four climate variables. Predicting *P*, the probability of finding a specimen in a site, the model is:
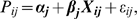
(1)where *P_ij_* is the probability of seeing plant *i* of species *j* at a site given ***α_j_*** is the intercept for species *j* and ***β_j_X_ij_*** is the design matrix of climate variables and their coefficients. The error term, *ε_ij_*, is distributed as a logistic random variable with set variance of 1.6 [33]. The intercept and all first order regression coefficients then have their own regression equations at the species-level of the model:
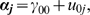
(2a)and
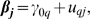
(2b)where *γ_00_* and *γ_0q_* are the intercepts for the species intercepts and the *q* in 1, …, *Q* first order regression coefficients (the four climate variables). In these species-level models, the residuals error terms *u_0j_* and *u_qj_* are distributed normal with mean 0 and variance *τ_0_* and *τ_q_* respectively. Because these regression models are estimated simultaneously and iteratively by weighting the information both within and across species, the combined model is an unbiased estimate of the regression coefficients of primary interest, ***α*** and ***β***. The estimation was done using penalized quasi-likelihood (PQL) method in the lme4 package [68].

For each individual species, the model estimates random parameters for linear versions of the intercept and the four climate variables. The model estimates all parameters simultaneously, and the structure of the model allows poorly known species to draw strength from the rest of the flora. Effectively, this causes poorly known species to behave more like the average of the flora. The individual influence of error prone, poorly known species is thus appropriately weighted in diversity maps for the entire flora.

### Future climate data

The future climate simulations are from the U.K. Meteorological Office Hadley Climate Centre Model version 3 (HadCM3) [23]–[24] and the DOE/NCAR Parallel Climate Model (PCM) [25] general circulation models (GCMs). We used these simulations to generate projections of future changes in temperature and precipitation over the region of interest. HadCM3 is a mid-high sensitivity model that produces a greater temperature response to a given amount of greenhouse gas emissions than does PCM, a low-sensitivity model. To project future emissions from human activities, we used the SRES higher (A1FI) and lower (B1) emissions scenarios that capture to some extent the uncertainty in future climate due to human decisions [22], with CO_2_ emissions ranging from slightly less than present-day levels up to four times present-day levels by 2100. Our climatological future time period represents 80 years (average of 2070–2099) from now.

The HadCM3 and PCM simulations project increases in mean annual temperatures averaged across the state of California of 2.3–2.2°C under B1 and 3.8–5.8°C under A1FI by 2070–2099. The models also project increases in the magnitude of seasonal temperature differences in most areas. Rainfall predictions are more variable among models. Changes range from decreases of 157 mm to increases of 38 mm of total annual precipitation. Within the United States, the global climate outputs were statistically downscaled to 1/8th-degree resolution [3]. Slight discontinuities along the US-Mexico border result primarily from downscaling discrepancies in precipitation estimates. From these data, we obtained four near-term and four long-term future climate scenarios by adding the differential between future time periods and the baseline time period for each model and emission scenario to each current monthly baseline climate map. Future climates were then projected into the two PCA spaces as passive variables to obtain future values for the four axes representing temperature and precipitation (see Figs. S6 through S7).

## Supporting Information

Figure S1 Histograms of the density of species centroid shifts in kilometers for each climate change scenario. (A–D) Scenarios in which species are permitted to move. (E–H) Scenarios in which species are not permitted to move. (A, B, E, F) Climate simulated by the PCM model. (C, D, G, H) Climate simulated by the HadCM3 model. (A,C,E,G) Scenarios with B1 emission levels. (B,D,F,H) Scenarios with A1FI emission levels.
(7.17 MB TIF)

Figure S2 Density histograms of mean elevation of species ranges in the present (blue) and future (red) for each climate change scenario. (A–D) Scenarios in which species are permitted to move. (E–H) Scenarios in which species are not permitted to move. (A, B, E, F) Climate simulated by the PCM model. (C, D, G, H) Climate simulated by the HadCM3 model. (A,C,E,G) Scenarios with B1 emission levels. (B,D,F,H) Scenarios with A1FI emission levels.
(3.89 MB TIF)

Figure S3 Directional histograms of species centroid movement for selected scenarios. Histograms are overlaid for different elevational zones, based on the species present elevation. The length of the vector in each direction is the percent of the corresponding flora that moves in that direction based on 591 species modeled with Maxent. (A) Climate simulated by the HadCM3 model with A1FI emission levels (severe scenario) where species are not permitted to move. (B) Climate simulated by the PCM model with B1 emission levels (less severe scenario) where species are not permitted to move. (C) Climate simulated by the PCM model with B1 emission levels (less severe scenario) where species are permitted to move.
(13.49 MB TIF)

Figure S4 Distributions of range size changes across all scenarios grouped by 6 range size change categories. (A–D) Scenarios in which species are permitted to move. (E–H) Scenarios in which species are not permitted to move. (A, B, E, F) Climate simulated by the PCM model. (C, D, G, H) Climate simulated by the HadCM3 model. (A,C,E,G) Scenarios with B1 emission levels. (B,D,F,H) Scenarios with A1FI emission levels.
(10.89 MB TIF)

Figure S5 (A–D) Current climate layers derived from PCA analyses and (E–H) corresponding climate variables. (A) Temperature magnitude (Axis 1 of a PCA of monthly mean temperature representing 69% of variation). (B) Temperature seasonality (Axis 2 of a PCA of monthly mean temperature representing 20% of variation). (C) Precipitation magnitude (Axis 1 of a PCA of monthly total precipitation representing 48% of variation). (D) Precipitation seasonality (Axis 2 of a PCA of monthly total precipitation representing 21% of variation). (E) Mean annual temperature ({degree sign}C). Correlation with Temperature Axis 1 is 1.000. (F) Standard Deviation of mean monthly temperatures ({degree sign}C). Correlation with Temperature Axis 2 is 0.998. (G) Total Annual Precipitation (cm). Correlation with Precipitation Axis 1 is 0.980. (H) Coefficient of variation of total monthly precipitation (cm). Correlation with Precipitation Axis 2 is 0.673.
(13.28 MB TIF)

Figure S6 Projected change in temperature magnitude (Axis 1, arbitrary units) (A–D) and temperature seasonality (Axis 2, arbitrary units) (E–H) under future climate change scenarios. (A, E) PCM B1. (B, F) PCM A1FI. (C, G) HadCM3 B1. (D, H) HadCM3 A1FI.
(19.98 MB DOC)

Figure S7 Projected change in precipitation magnitude (Axis 1, arbitrary units) (A–D) and precipitation seasonality (Axis 2, arbitrary units) (E–H) under future climate change scenarios. (A, E) PCM B1. (B, F) PCM A1FI. (C, G) HadCM3 B1. (D, H) HadCM3 A1FI.
(19.89 MB TIF)

Table S1 Distribution data for each endemic species. The first two columns list the TJM1 range sizes in sq. kilometers and the number of specimens. The next two columns indicate whether the species was modeled with Maxent (>41 specimens) and included in the MLGLM (>1 specimens). The last column indicates the number of randomly selected informed pseudo-absences for use in the MLGLM.
(0.27 MB XLS)

Table S2 Maxent model performance for the 591 best known species. The first two columns list the number of specimens used to test and train the models. The next column lists the area under the receiver operating characteristic curve (AUC) evaluation statistic which ranges from 0.5 to 1. The next column lists the threshold used to create binary ranges from the cumulative index ranging from 0 to 100. The last column lists the prediction success evaluation statistic which is the percent of test specimens correctly predicted by the binary ranges.
(0.10 MB XLS)
