# Supplementary figures and images for: Climate Change and the Future of California's Endemic Flora
Source: PLoS One. 2008 Jun 25;3(6):e2502. doi: 10.1371/journal.pone.0002502 (PMC2481286; doi:10.1371/journal.pone.0002502)

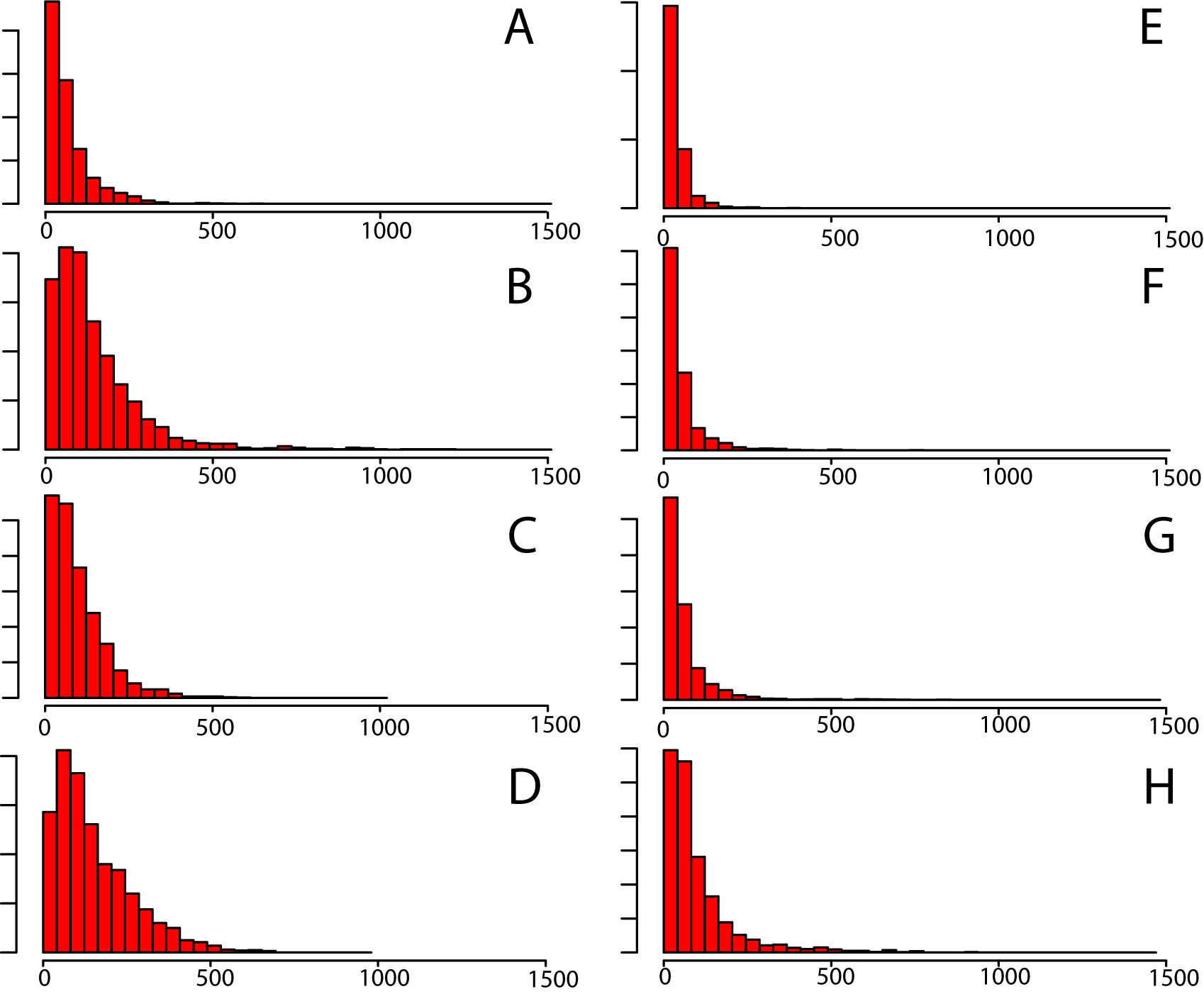

Supplement: Figure S1 — Histograms of the density of species centroid shifts in kilometers for each climate change scenario. (A–D) Scenarios in which species are permitted to move. (E–H) Scenarios in which species are not permitted to move. (A, B, E, F) Climate simulated by the PCM model. (C, D, G, H) Climate simulated by the HadCM3 model. (A,C,E,G) Scenarios with B1 emission levels. (B,D,F,H) Scenarios with A1FI emission levels. (7.17 MB TIF) [file pone.0002502.s001.tif]

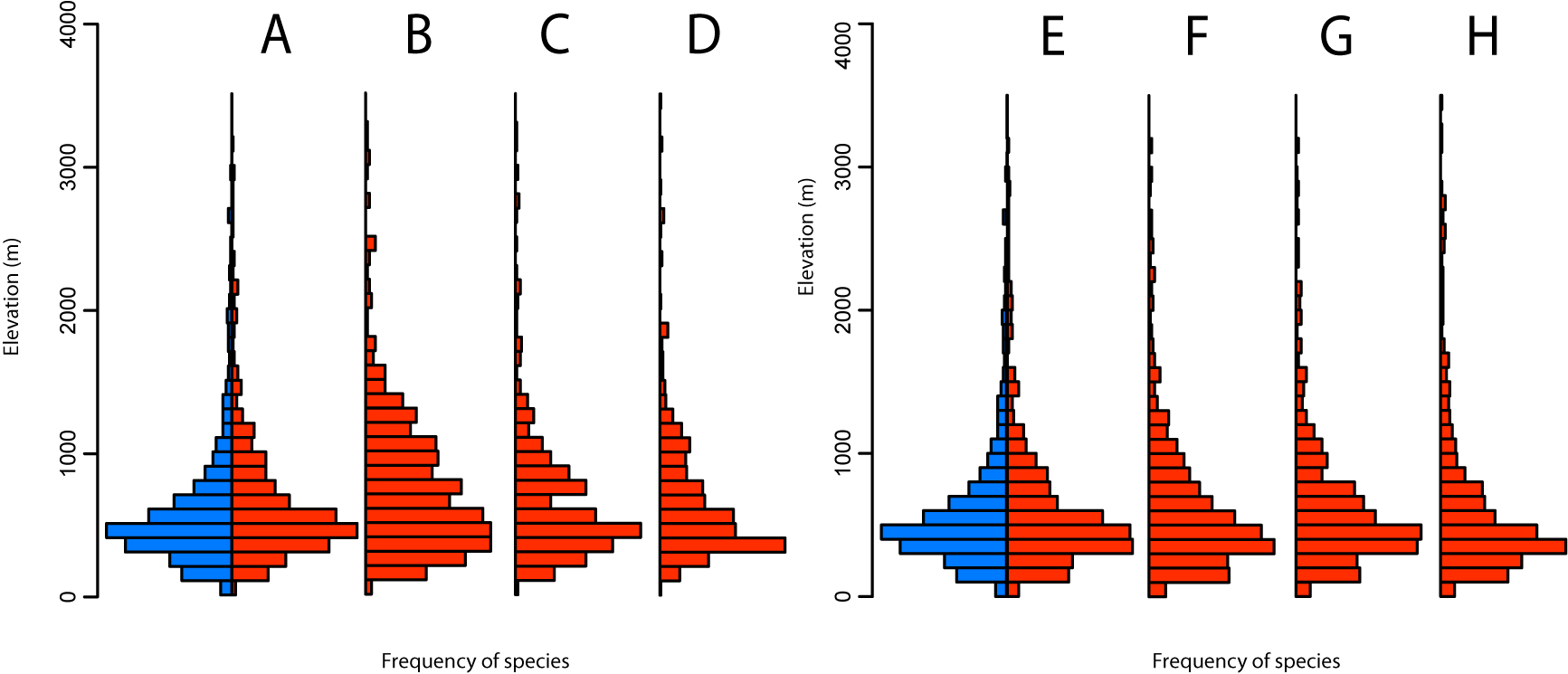

Supplement: Figure S2 — Density histograms of mean elevation of species ranges in the present (blue) and future (red) for each climate change scenario. (A–D) Scenarios in which species are permitted to move. (E–H) Scenarios in which species are not permitted to move. (A, B, E, F) Climate simulated by the PCM model. (C, D, G, H) Climate simulated by the HadCM3 model. (A,C,E,G) Scenarios with B1 emission levels. (B,D,F,H) Scenarios with A1FI emission levels. (3.89 MB TIF) [file pone.0002502.s002.tif]

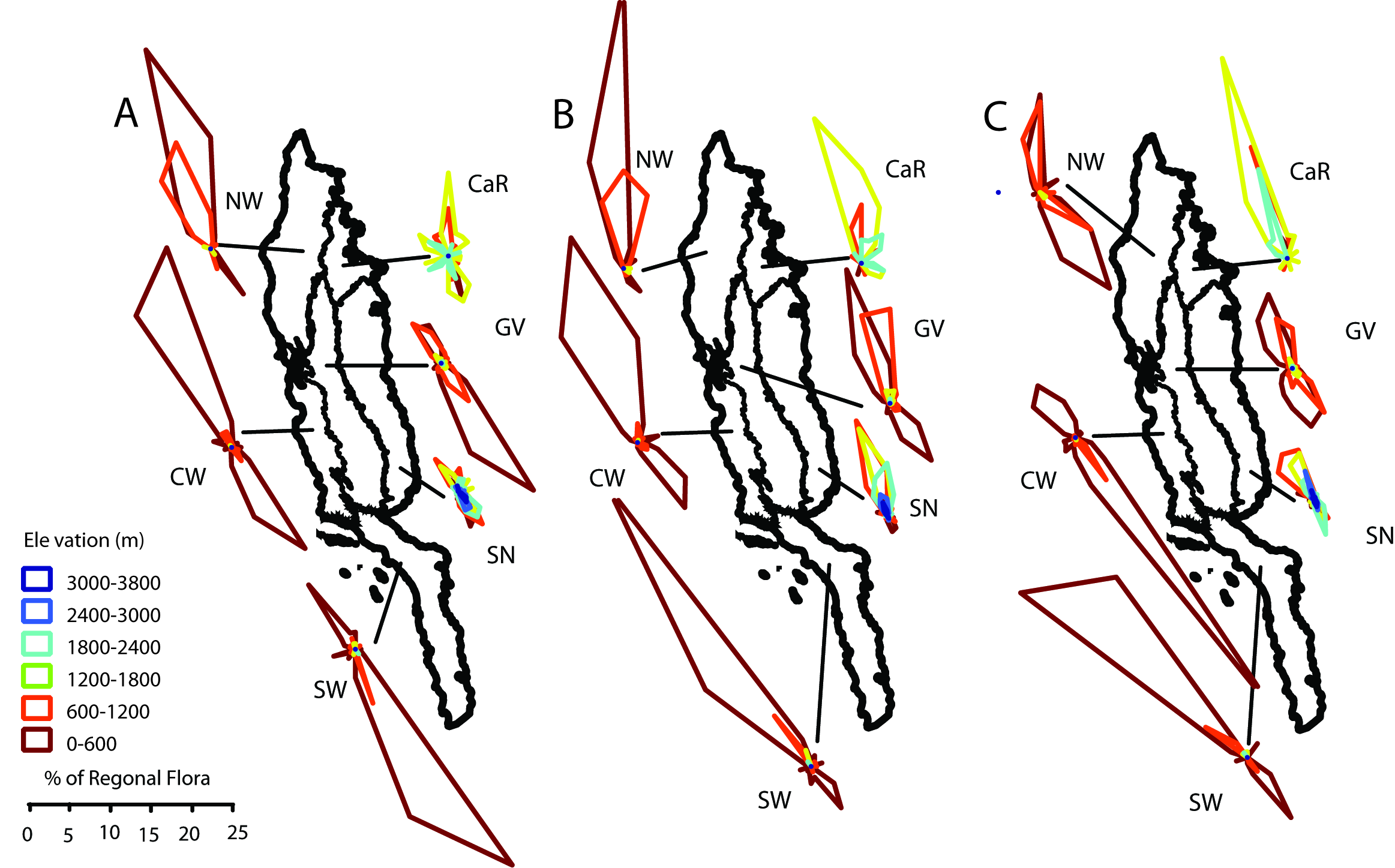

Supplement: Figure S3 — Directional histograms of species centroid movement for selected scenarios. Histograms are overlaid for different elevational zones, based on the species present elevation. The length of the vector in each direction is the percent of the corresponding flora that moves in that direction based on 591 species modeled with Maxent. (A) Climate simulated by the HadCM3 model with A1FI emission levels (severe scenario) where species are not permitted to move. (B) Climate simulated by the PCM model with B1 emission levels (less severe scenario) where species are not permitted to move. (C) Climate simulated by the PCM model with B1 emission levels (less severe scenario) where species are permitted to move. (13.49 MB TIF) [file pone.0002502.s003.tif]

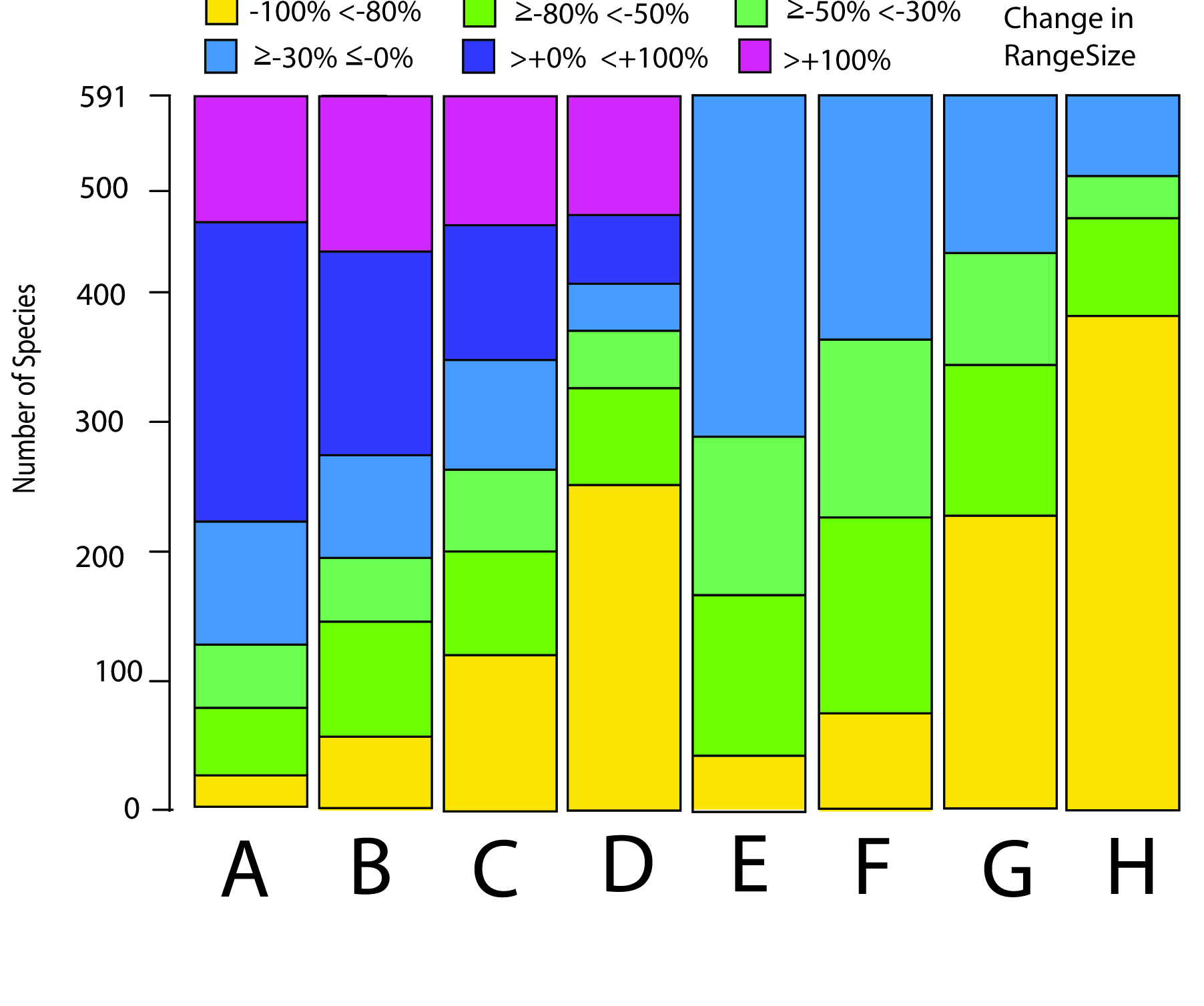

Supplement: Figure S4 — Distributions of range size changes across all scenarios grouped by 6 range size change categories. (A–D) Scenarios in which species are permitted to move. (E–H) Scenarios in which species are not permitted to move. (A, B, E, F) Climate simulated by the PCM model. (C, D, G, H) Climate simulated by the HadCM3 model. (A,C,E,G) Scenarios with B1 emission levels. (B,D,F,H) Scenarios with A1FI emission levels. (10.89 MB TIF) [file pone.0002502.s004.tif]

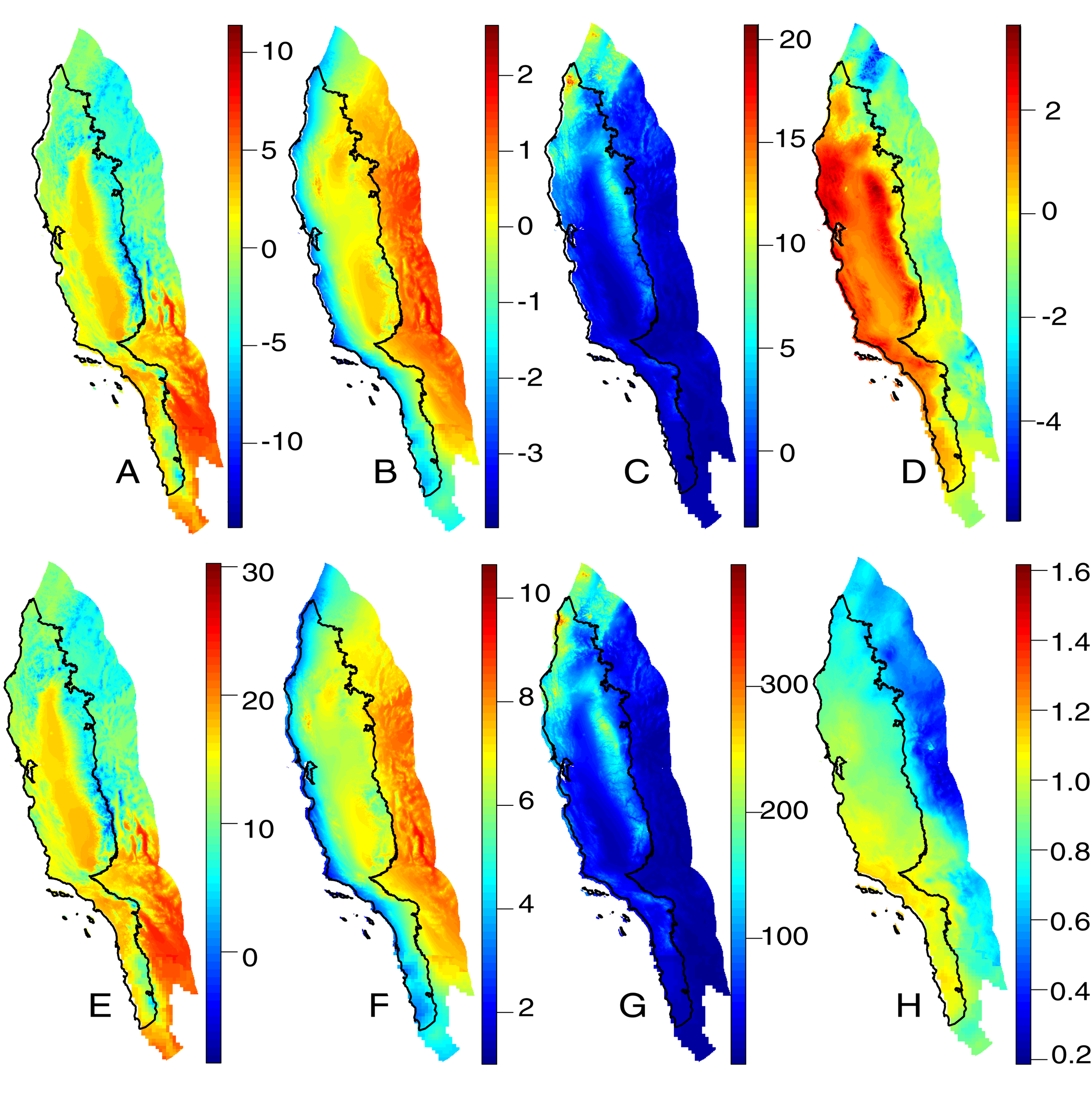

Supplement: Figure S5 — (A–D) Current climate layers derived from PCA analyses and (E–H) corresponding climate variables. (A) Temperature magnitude (Axis 1 of a PCA of monthly mean temperature representing 69% of variation). (B) Temperature seasonality (Axis 2 of a PCA of monthly mean temperature representing 20% of variation). (C) Precipitation magnitude (Axis 1 of a PCA of monthly total precipitation representing 48% of variation). (D) Precipitation seasonality (Axis 2 of a PCA of monthly total precipitation representing 21% of variation). (E) Mean annual temperature ({degree sign}C). Correlation with Temperature Axis 1 is 1.000. (F) Standard Deviation of mean monthly temperatures ({degree sign}C). Correlation with Temperature Axis 2 is 0.998. (G) Total Annual Precipitation (cm). Correlation with Precipitation Axis 1 is 0.980. (H) Coefficient of variation of total monthly precipitation (cm). Correlation with Precipitation Axis 2 is 0.673. (13.28 MB TIF) [file pone.0002502.s005.tif]

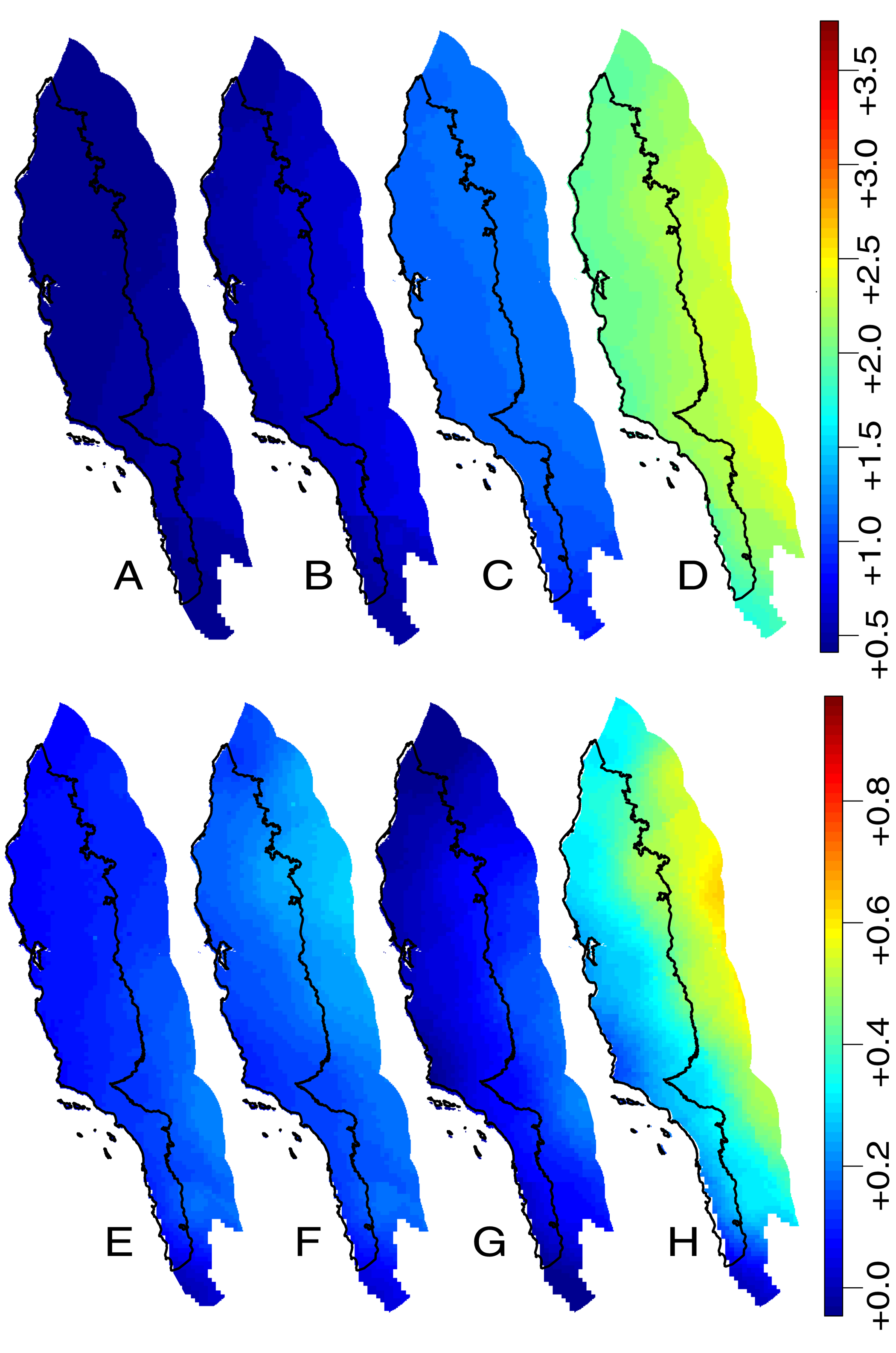

Supplement: Figure S6 — Projected change in temperature magnitude (Axis 1, arbitrary units) (A–D) and temperature seasonality (Axis 2, arbitrary units) (E–H) under future climate change scenarios. (A, E) PCM B1. (B, F) PCM A1FI. (C, G) HadCM3 B1. (D, H) HadCM3 A1FI. (19.98 MB DOC) [file pone.0002502.s006.tif]

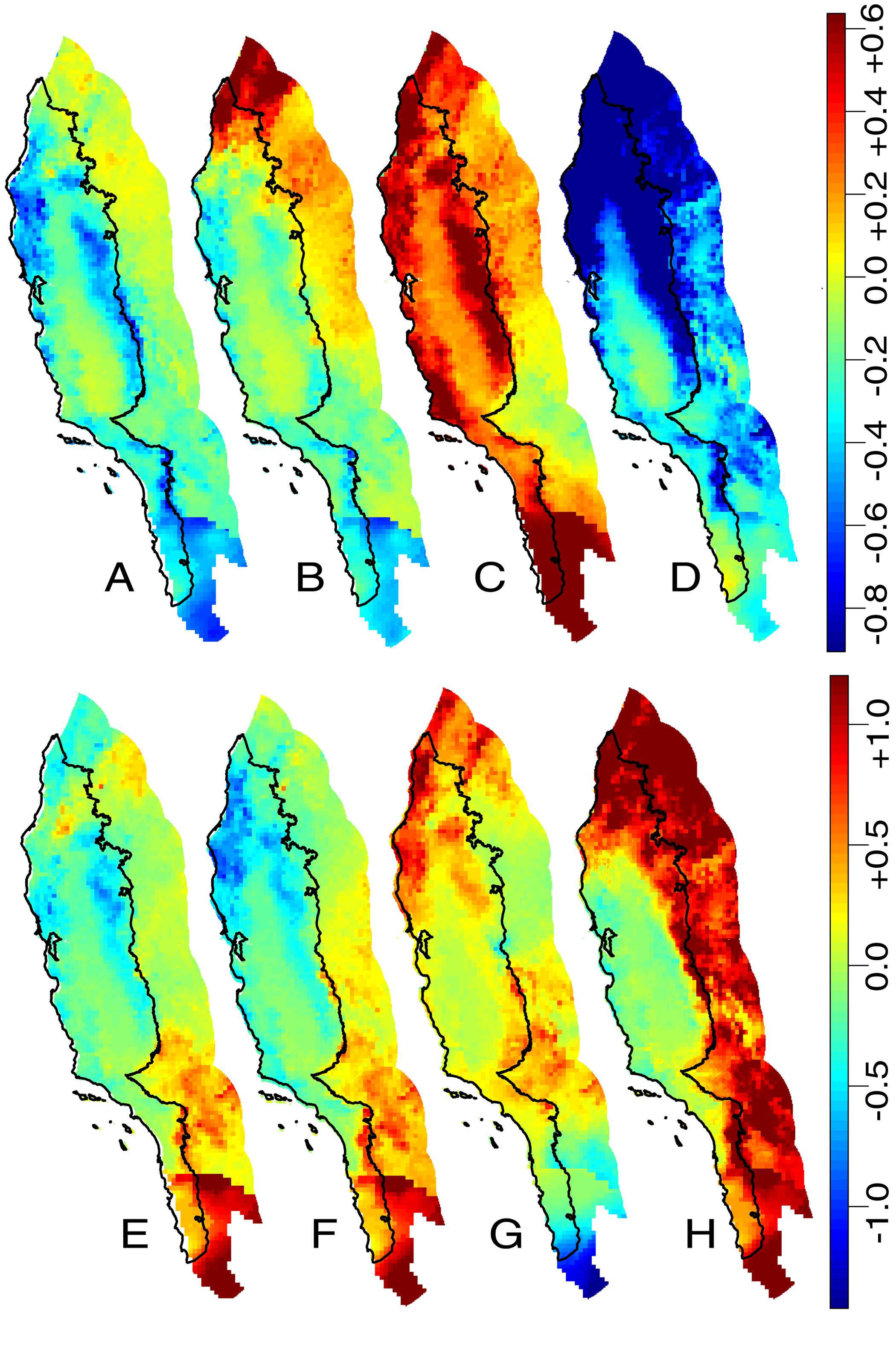

Supplement: Figure S7 — Projected change in precipitation magnitude (Axis 1, arbitrary units) (A–D) and precipitation seasonality (Axis 2, arbitrary units) (E–H) under future climate change scenarios. (A, E) PCM B1. (B, F) PCM A1FI. (C, G) HadCM3 B1. (D, H) HadCM3 A1FI. (19.89 MB TIF) [file pone.0002502.s007.tif]
